# Effect of Hydraulic Retention Time on Anaerobic Digestion of Wheat Straw in the Semicontinuous Continuous Stirred-Tank Reactors

**DOI:** 10.1155/2017/2457805

**Published:** 2017-05-14

**Authors:** Xiao-Shuang Shi, Jian-Jun Dong, Jun-Hong Yu, Hua Yin, Shu-Min Hu, Shu-Xia Huang, Xian-Zheng Yuan

**Affiliations:** ^1^State Key Laboratory of Biological Fermentation Engineering of Beer, Tsingtao Brewery Co., Ltd, Qingdao 266100, China; ^2^Shandong Industrial Engineering Laboratory of Biogas Production & Utilization, Key Laboratory of Biofuels, Qingdao Institute of Bioenergy and Bioprocess Technology, Chinese Academy of Sciences, Qingdao, Shandong Province 266101, China

## Abstract

Three semicontinuous continuous stirred-tank reactors (CSTR) operating at mesophilic conditions (35°C) were used to investigate the effect of hydraulic retention time (HRT) on anaerobic digestion of wheat straw. The results showed that the average biogas production with HRT of 20, 40, and 60 days was 46.8, 79.9, and 89.1 mL/g total solid as well as 55.2, 94.3, and 105.2 mL/g volatile solids, respectively. The methane content with HRT of 20 days, from 14.2% to 28.5%, was the lowest among the three reactors. The pH values with HRT of 40 and 60 days were in the acceptable range compared to that with HRT of 20 days. The propionate was dominant in the reactor with HRT of 20 days, inhibiting the activities of methanogens and causing the lower methane content in biogas. The degradation of cellulose, hemicellulose, and crystalline cellulose based on XRD was also strongly influenced by HRTs.

## 1. Introduction

The estimated annual production of wheat straw is 681.92 million tons of dry biomass, produced as a by-product of wheat cultivation throughout the world [1]. Although a part of the wheat straw is utilized as feed for animals, the major part remains undisposed as environment pollutants or burnt in field [2]. In China, the utilization of wheat straw for renewable energy is increasing gradually due to the greenhouse gas reduction and renewable energy demand [3]. The conversion of wheat straw to renewable energy could be achieved in many ways. And biogas production through anaerobic digestion is considered ideal due to the economic benefit and energy efficiency [4, 5].

Anaerobic digestion is a complex biochemical process, whereby microbes decompose organic matter and produce biogas. A number of parameters could influence the performance and biogas production for semicontinuous or continuous anaerobic digestion [1], including substrate characteristics, organic loading rate (OLR), hydraulic retention time (HRT), temperature, and pH. HRT is an important operational parameter for the anaerobic reactors which can affect the conversion of volatile solids (VS) into biogas [6, 7]. Generally, relatively long HRT is needed in anaerobic digestion of lignocellulosic wastes for this type of substrates is persistent to anaerobic microbes [8]. Rivard et al. [9] suggested that 60–90 days is required in order to achieve complete digestion of polymeric substrates, while Banks [10] also reported HRT of 20 days in anaerobic digestion of maize. Shorter HRT is desirable as it is directly related to the reduction of capital cost and the increase of process efficiency. However, literature is still lacking in the effects of HRT on anaerobic digestion of wheat straw, the typical lignocellulosic wastes.

The main objective of this research was to identify the effect of HRT on anaerobic digestion of wheat straw in the semicontinuous continuous stirred-tank reactors (CSTR). The biogas production, methane content, pH value, and volatile fatty acids (VFAs) component were selected as the main evaluated factors to study the influence of HRT. In addition, the degradation of cellulose, hemicellulose, and crystalline cellulose in digested wheat straw was also evaluated.

## 2. Materials and Methods

### 2.1. Substrate and Inoculum

The sun-dried wheat straw used in this study was donated by Dr. Yongbao Chu (College of Environment and Safety Engineering, Qingdao University of Science and Technology, China) and milled to 40-mesh powder as described previously [11]. Inoculum was originated from a 1000-m^3^ size of sludge digestion tank (Qingdao, China) operating at 35°C, with a 20-day retention time. The total solid (TS) and volatile solid (VS) contents were 30.2% and 55.3% TS, respectively.

### 2.2. Anaerobic Digestion

The experiments were carried out in three semicontinuous CSTR with the working volume of 4.0 L, which was covered with 0.30-mm stainless steel meshes, fabricated from 10-mm polymethylmethacrylate sheets. With a temperature-controlled water bath at 35°C, the reactor was connected to a wet-type gas flow meter and gas sampling ports using silicone tubes. At the beginning, all digesters were inoculated and set in batch mode fermentation until the stability of biogas production. Afterwards, each digester was fed with a TS concentration of 8% at HRT of 20, 40, and 60 days, respectively. The durations of the reactors were one HRT time for different reactors.

### 2.3. Analytical Methods

The daily biogas production was recorded by the gas flow meter. Samples from the digester were daily collected for measurements of pH, biogas component, and VFAs. Biogas main components including hydrogen, methane, and carbon dioxide were analyzed by a gas chromatograph (SP 6890, Shandong Lunan Inc., China) equipped with Porapak Q stainless steel column (180 cm long, 3 mm outer diameter) and a thermal conductivity detector. The injector, detector, and oven temperatures were 120°C, 150°C, and 50°C, respectively. VFAs were measured by a gas chromatograph (450-GC, Varian, USA), equipped with Innowax column (30 m ×  Ф 0.25 mm × 0.25 *μ*m) and flame ionization detectors when the samples were filtered through a 0.45-*μ*m GF/C grade binder free glass microfiber filter. The operating temperatures were 220°C, 250°C, and 150°C for injection port, detector, and oven, respectively. The content of cellulose, hemicellulose, and lignin was estimated as described previously [12]. TS, VS, ammonia, and pH value were determined according to the standard methods of “Monitor and Analysis Method of Water and Wastewater. Chinese Environmental Science Publication” [13].

### 2.4. X-Ray Diffractometry

X-ray diffraction (XRD) was performed with a D5 advancer diffractometer as described before [11] with Ni-filtered Cu K_*α*_ radiation at a wavelength of 0.1541 nm operated at 40 kV and 200 mA. Crystallinity of cellulose (CrI: the crystalline index) was afterwards calculated using the formula developed by Kim et al. [14]:

(1) $$\begin{array}{c} CrI\left(\;\%\;\right)=\frac{I_{002}-I_{18.0^{{}^{\circ}}}}{I_{002}}\times 100. \end{array}$$

CrI is the crystalline index; *I*_002_ is the maximum intensity of the (002) lattice diffraction; *I*_18.0°_ is the intensity diffraction at 2*θ* degree of 18.0°.

## 3. Results and Discussion

### 3.1. Biogas Production and Methane Content

**Figure 1:**
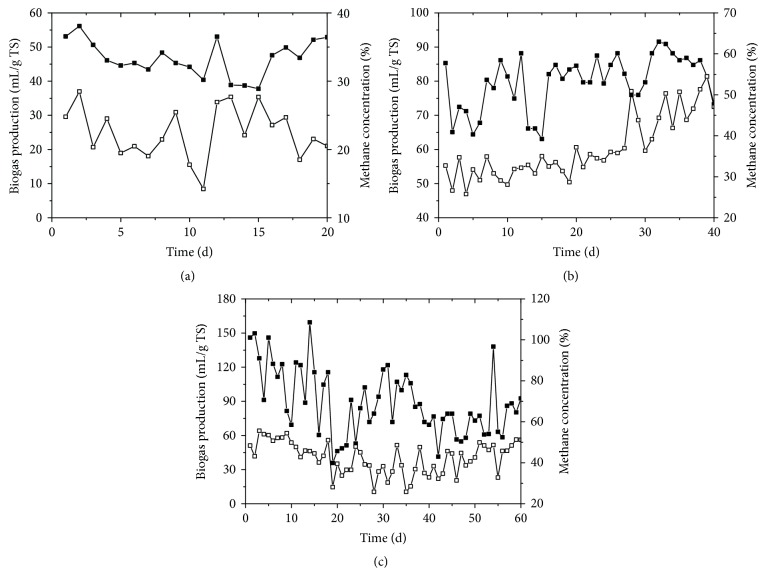
Biogas production (■) and methane concentration (□) in anaerobic digestion of wheat straw with HRT of 20 (a), 40 (b), and 60 (c) days.

The effects of HRT on anaerobic digestion of wheat straw in CSTR were conducted after all digesters reached steady-state. As shown in Figure 1, the biogas production was obviously affected by HRT. The average biogas production with HRT of 20, 40, and 60 days was 46.8, 79.9, and 89.1 mL/g TS as well as 55.2, 94.3, and 105.2 mL/g volatile solids, respectively. However, the variation range of daily biogas with HRT of 60 days was more greatly compared to those with HRT of 20 and 40 days, from 35.9 to 159.4 mL/g TS. Anaerobic digestion of wheat straw has been determined by various researchers. Weiland [15] presented the methane yield of wheat straw with 390 mL/g organic dry matter fed to the anaerobic digester. Gunaseelan [16] reported that the methane production from anaerobic digestion of wheat straw ranged from 190 to 327 mL/g VS. In addition, Møller et al. [17] reported the methane production was 145–161 mL/g VS wheat straw.

The effect of HRT on the methane content is also shown in Figure 1. The average methane contents with HRT of 20, 40, and 60 days were 22.4%, 36.9%, and 42.4%, respectively. The methane contents with HRT of 20, from 14.2% to 28.5%, were the lowest among three reactors. Generally, the methanogens have a long regeneration time compared with the hydrolysis acidogenesis bacteria. In order to avoid being washed out from the reactor, HRT must be long enough to retain the methanogens. For example, Ma et al. [18] indicated that the anaerobic sequential batch reactor treating a dilute waste stream was failure when the HRT was shorter than 2 days, for the HRT was too short to exceed the microorganism growth limits. Similarly, in this study, the longer HRT is, the more methane content was achieved.

### 3.2. Operation Stability

**Figure 2:**
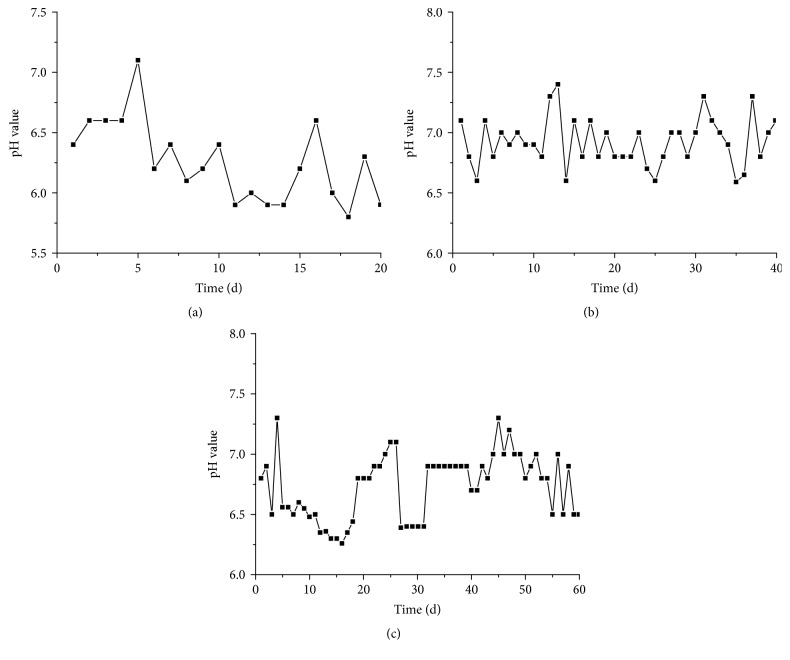
The pH value in anaerobic digestion of wheat straw with HRT of 20 (a), 40 (b), and 60 (c) days.

For anaerobic digestion, pH significantly affects the performance, especially for the lignocellulosic substrates. The effect of HRT on pH values during anaerobic digestion of wheat straw is shown in Figure 2. The pH values ranged from 5.8 to 7.1 with HRT of 20 days, while the pH values ranged from 6.6 to 7.4 and from 6.3 to 7.3 with HRT of 40 and 60 days, respectively. The optimum pH range for high-solid (4–10% TS) anaerobic digestion was 6.6–7.8, while the acceptable pH range was 6.1–8.3 [19]. The pH values with HRT of 40 and 60 days were in the acceptable range. However, the lowest pH value with HRT of 20 days was 5.8, which was not at a level appropriate for substrate degradation and was not supportive of the production of methane.

**Figure 3:**
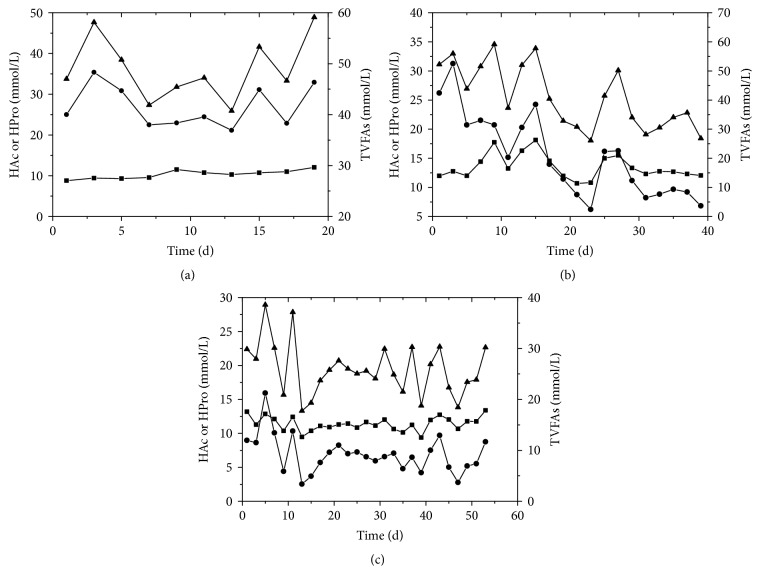
HAc (■), HPro (●), and TVFAs (▲) in anaerobic digestion of wheat straw with HRT of 20 (a), 40 (b), and 60 (c) days.

The effects of HRT on acetate (HAc), propionate (HPro), and TVFAs in anaerobic digestion of wheat straw are shown in Figure 3. The dominant VFAs in the reactor with HRT of 20 days were HPro, accounting for more than 50% of the TVFAs. And the HAc contents in this reactor were 8.8 to 12.0 mmol/L during the digestion period. For the reactor with HRT of 40 days, the HAc contents were lower than the HPro contents in the initial 20 days. Then the HPro contents decreased gradually and were lower than the HAc contents in the end of the digestion. However, the HAc was dominant in the reactor with HRT of 60 days, which ranged from 33.4% to 57.9% in the TVFAs. And the HPro contents were 2.5 to 15.9 mmol/L in this reactor and were lower than 30% of TVFAs. It is widely believed that propionate could cause greater inhibition to the activity of methanogens than other VFAs due to the fact that propionate biodegradation pathway involves unusual and complicated enzyme reactions [20]. Wong et al. [21] fed the digester with a pure propionate in a 2.5 L UASB reactors and found that the propionate exhibited severe inhibition, leading to system failure at a feed concentration of 20 g COD/L. The HPro was the dominated species VFAs in the reactor with HRT of 20 days, which might inhibit the activities of methanogens and cause the low methane content in biogas.

### 3.3. Substrate Degradation

In order to analyze the effect of HRT on the substrate degradation, the contents of cellulose, hemicellulose, and lignin of in effluent were determined three times during the whole process with different HRTs. The cellulose and hemicellulose in wheat straw can hydrolyze into glucose and xylose, respectively, which are able to be converted to biogas. In addition, the lignin is hardly degraded during anaerobic digestion [22, 23]. In this study, it was assumed that the lignin was not degraded during the whole process. Hence, the degradations of cellulose and hemicellulose are shown in Table 1. From Table 1, degradations of cellulose and hemicellulose with HRT of 20 days were 43.8% and 47.1%, respectively, lowest among three reactors. This was consistent with the biogas production. The degradations of cellulose with HRT of 40 and 60 days were 52.1% and 55.4%, respectively, along with the hemicellulose degradation of 71.4% and 76.8%, respectively.

**Figure 4:**
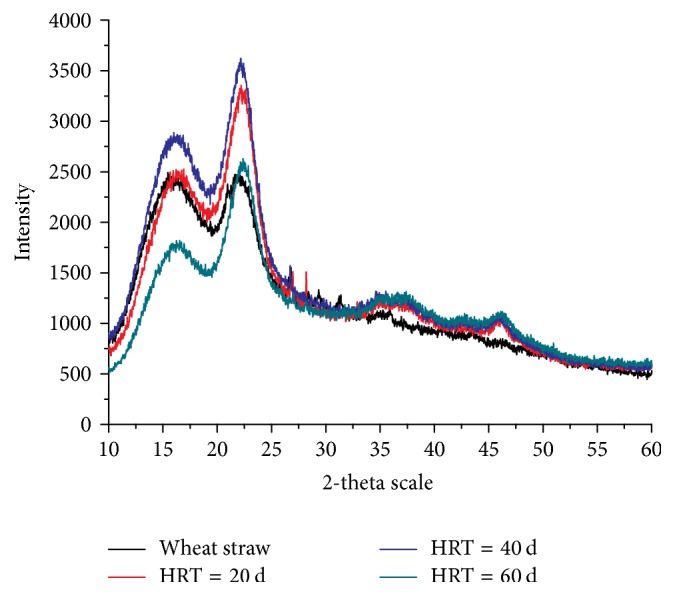
X-ray diffraction patterns of wheat straw and digested wheat straw with different HRTs.

Cellulose in wheat straw is a complex polymer with both crystalline and amorphous areas [24]. From the X-ray diffraction patterns of digested wheat straw with different HRTs (shown in Figure 4), the CrI of digested straw was strongly influenced by HRTs. The CrI dropped from 46.4% to 30.9%, 24.7%, and 25.9% with HRT of 20, 40, and 60 days, respectively. For lignocellulosic biomass, the CrI indicates the relative amount of crystalline cellulose in the substrates. Hence, the above data presented that part of crystalline cellulose was disrupted and longer HRT leads to higher disruption. Pan et al. [25] showed that the CrI of acid-treated cellulose decreased from 64.1% to 60.4% after hydrogen fermentation. However, the CrI of nonpretreated corncob dropped from 48.3% to 44.3%, which was lower than that of this study.

## 4. Conclusions

Effects of HRT on anaerobic digestion of wheat straw in semicontinuous CSTR were investigated. The results showed that HRT could influence the biogas production, methane content, pH value, and VFA component. The average biogas production with HRT of 20, 40, and 60 days was 55.2, 94.3, and 105.2 mL/g volatile solids, respectively. The digestion with HRT of 20 days showed lower stability compared with those with HRT of 40 and 60 days. The degradations of cellulose with HRT of 20, 40, and 60 days were 43.8%, 52.1%, and 55.4%, respectively, along with the hemicellulose degradation of 47.1%, 71.4%, and 76.8%, respectively. All the results indicated that HRT is an important parameter to influence the performance and stability in anaerobic digestion of wheat straw.

## Figures and Tables

**Table 1:** The content and degradation of cellulose, hemicellulose, and lignin in the effluent with different HRTs.

HRT (d)		20	40	60
Cellulose	Content (% TS)	36.2	32.5	32.7
	Degradation (%)	43.8	52.1	55.4
Hemicellulose	Content (% TS)	18.4	10.5	9.2
	Degradation (%)	47.1	71.4	76.8
Lignin	Content (% TS)	9.4	9.9	10.7
